# Assessing the knowledge, attitude, and practice of frontline physicians in Egyptian university hospitals regarding pharyngitis and acute rheumatic fever: a cross-sectional study that calls for action

**DOI:** 10.1186/s12889-024-19658-5

**Published:** 2024-08-16

**Authors:** Kerollos Abdelsayed, Hossam Tharwat Ali, Mohamed Basyouni Helal, Ahmed Assar, Maysa Madany, Mohamed Diaa Gabra, Ahmed Abdelrahman, Yomna Goudy, Ahmed Dandrawy, Ziad Ashraf Soliman, Heba M. Qubaisy, Ghada. M. M. Shahin, ARF –RHD Prevention Group, Mohamed Elsayed Saleh, Aiman Al-Touny, Wael Reda Attallah Soliman, Mohamed Omer, Mohannad Ahmed Hassan Mahmoud, Ola Youssef, Ali Noshey Abdelaziz Abdelrahim, Esraa Y. Salama, Mostafa Elfrly, Baraa Muthanna Ali, Mariam A. Shaltout, Abdelhamid Salah Abdelhamid Abdelrahim, Mohamed Elbahnasawy, Mohammed Ayman Mohammed, Basma Akram Mohamed, Modather Moharam, Ammar Yasser Negm, Haya Mohamed, Shymaa Mohamed Abo Ghanimaa, Shimaa A. Al-Touny, Mahmoud M. Saad, Seif Elnamas, Youssef Farag, Tasneem Abdelrhman ElsayedElsayed, Kyrillos Mahrous Gerges, Emad M. Hammad, Eman Gamal Esmail Isawy, Noor Hossameldeen Abdelaziz, Parvin C. Azimullah

**Affiliations:** 1 Present Address: Aswan Heart Centre, Magdi Yacoub Foundation, Aswan, Egypt; 2 Qena Faculty of Medicine, South Valley University, Qena, 83523 Egypt; 3 Clinical Research Department, Qena Student Research Association, Qena, Egypt; 4 Faculty of Medicine, Menofia University, Menofia, Egypt; 5 Department of Pediatrics, Qena Faculty of Medicine, South Valley University, Qena, Egypt; 6 Department of Cardiothoracic Surgery, Leiden University Medical Center, Albinusdreef 2, Leiden, 2333 ZA the Netherlands; 7 Occupational Health Physician, PCA Medical Consultancy, Christianiastraat 2, Haarlem, 2034KB the Netherlands; 8 Present Address: Department of Rheumatology and Immunology, Qena General Hospital, Qena, Egypt

**Keywords:** Acute rheumatic fever, Rheumatic heart disease, Pharyngitis, Knowledge, Attitude, Practice

## Abstract

**Background:**

Acute rheumatic fever (ARF) and rheumatic heart disease (RHD) remain major public health issues. Although the primary and secondary prevention of RHD through appropriate management of bacterial pharyngitis and ARF are well-described in the literature, few studies address the knowledge, attitude, and practice (KAP) of developing countries. We aimed to evaluate the KAP of the frontline physicians in Egyptian university hospitals regarding pharyngitis and ARF.

**Methods:**

We employed a cross-sectional design between September 1st, 2022, and January 31st, 2023 using a self-administered questionnaire in 21 Egyptian universities. The questionnaire was developed based on previous studies and recent guidelines and included four domains: sociodemographic data, knowledge, attitude, and practice regarding pharyngitis and ARF. We utilized both online (Google Forms) and paper surveys. Frontline physicians, including interns, residents, and assistant lecturers, were conveniently invited to participate. Furthermore, with the help of participating phycisians in recruiting their colleagues, we utilized the snowball method. Data were analyzed using IBM SPSS version 27 software.

**Results:**

The final analysis included 629 participants, of whom 372 (59.1%) were males and 257 (40.9%) had direct contact with ARF patients. Most participants (61.5%) had a fair knowledge level while 69.5% had a fair level of practice regarding ARF and pharyngitis. Higher satisfactory knowledge levels were noted regarding pharyngitis (17.1% vs. 11.3%; *p*-value: 0.036) and ARF (26.8% vs. 18%; *p*-value: 0.008) among physicians dealing directly with ARF cases compared to physicians in departments not dealing directly with ARF cases. Physicians in Cairo region universities had significantly higher levels of satisfactory knowledge about ARF compared to Delta and Upper Egypt region universities (*p* = 0.014). Delta region universities showed significantly lower levels of practice compared to Cairo and Upper Egypt region universities (*p* = 0.027). The most frequently recognized barriers against health promotion were low socioeconomic status (90.3%) and lack of adequate public education (85.8%).

**Conclusions:**

Despite the fair knowledge and practice levels towards bacterial pharyngitis and ARF among participants, many gaps were still identified that might contribute to RHD prevalence. Educational interventions should be implemented by updating the local guidelines in Egypt for diagnosis and management based on the most recent guidelines.

**Supplementary Information:**

The online version contains supplementary material available at 10.1186/s12889-024-19658-5.

## Introduction

Rheumatic Heart Disease (RHD) is the most common cause of acquired heart failure in patients younger than 25 years in developing countries [1]. An autoimmune response to group A streptococcal (GAS) pharyngitis leads to Acute Rheumatic Fever (ARF) which in turn causes RHD in 60% of cases [2, 3].

RHD affected 33.4 million people worldwide in 2019 leading to 305,651 deaths, with a mortality rate of of 9 per 1000, mostly in low and middle-income countries [3, 4]. According to the Egyptian Ministry of Health’s 2018 report, Egypt had 300,000 RHD patients of 5**–**15 years [2, 5]. An echocardiography-based study on 3062 schoolchildren aged 5–15 years revealed a prevalence of 31 cases per 1000 children [6]. The latest World Health Organization (WHO) data show a mortality from RHD in Egypt of 1248 deaths accounting for 0.23% of the total deaths [7]. This heavy burden of RHD contrasts with persistent negligence of the condition on nationwide health agendas in endemic countries [3, 4].

Many factors such as house overcrowding and low socio-economic status can accelerate the spread of GAS infection [1], leading to a higher prevalence of ARF and RHD [5]. Primary prevention of RHD relies mainly on the accurate diagnosis and management of ARF [8]. Although the Egyptian Ministry of Health and Population together with WHO-Egypt initiated the National RHD Prevention and Control Program in 2006 to emphasize proper practices for pharyngitis, ARF, and their sequelae [9], the knowledge level of physicians has not been investigated.

This study aims to assess the current knowledge level of pharyngitis and ARF amongst university fromtline healthcare providers in Egypt as well as factors impeding proper management and daily practice. The secondary objectives are to determine the correlation between knowledge, perception, and practice scores associated with demographic variables.

## Methods

### Study design and participants

A multicenter cross-sectional study was conducted in 21 universities in 15 cities in Egypt (Supplementary Table 1). The study was done between September 1st, 2022, and January 31st, 2023 using online and/or paper surveys. Frontline physicians in the university hospitals including interns (MBBCh), residents (MBBCh), and assistant lecturer physicians (MSc; specialists) of different departments were invited to participate in the study. Medical students were excluded. For the included physicians, subgrouping into department that are most likely to deal directly with pharyngitis and ARF cases included internal medicine, pediatrics, and ear, nose, and throat (ENT) while the others were deemed to be not dealing directly with such cases. The study adhered to the Strengthening The Reporting of Observational Studies in Epidemiology (STROBE) checklist in its entirety [10].

### Sampling and sample size calculation

Convenience sampling method was mainly used where the study collaborators efforted to distribute the survey in the social media groups of the physicians. Additionally, they visited the departments to invite the target physicians to participate in the survey. Due to the busy schedule of the university hospital physicians and lack of interest to participate in research surveys, we used the help of participating physicians to recruit their colleagues, implementing the snowball sampling method. The sample size was calculated using the following equation [*n* = z2P(1-P)/d2], confidence interval of 95%, expected frequency of 50%, and acceptable margin of error of 5%. The minimum sample size was 384 participants which was increased to 687 to increase the power of the study.

### Study tool and data collection

**Fig. 1:**
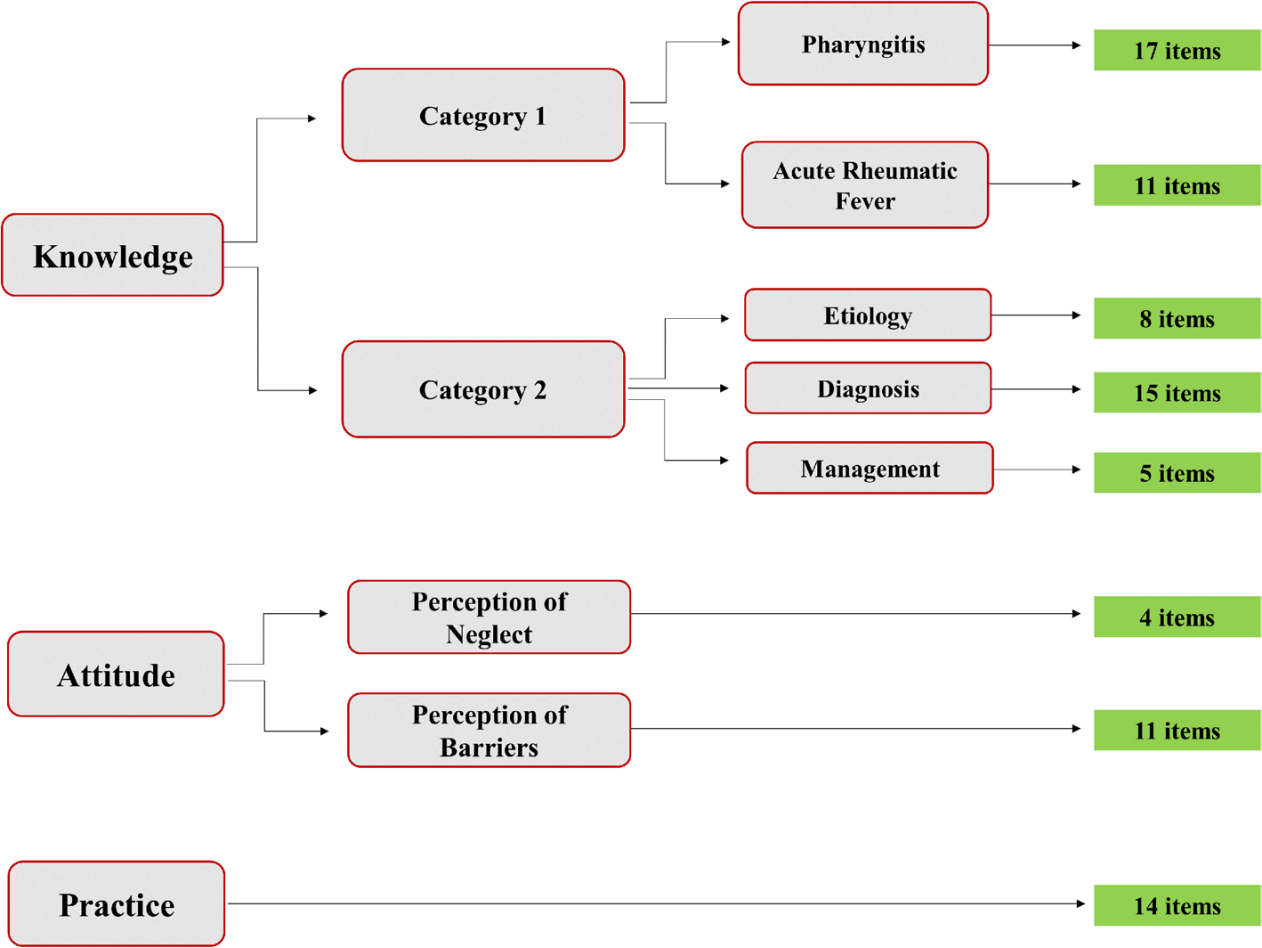
The structure of the questionnaire; domains, categories, subcategories, and questions

A self-administered Google Form survey in English was developed based on previous studies and recent guidelines [11–14]. It encompassed sociodemographic data and the domains of knowledge, attitude towards, and practices regarding pharyngitis and ARF. Sociodemographic data included gender, university, department, and medical degree. The survey domains, categories, subcategories, and number of questions are illustrated in Fig. 1. The complete survey is added in Supplementary Table 2.

At each university, authors and collaborators cooperated to manage, distribute, and collect the data. Consent from interns, residents, and assistant lecturer physicians to participate was asked. We excluded incomplete or double-filled-out surveys. Participants who participated in the pilot study were excluded from the final sample. The form was distributed through WhatsApp, LinkedIn, and emails. The link recorded the data anonymously. Those who could not access the online link were invited to complete a paper survey that was subsequently inserted into the study’s database.

### Validation and pilot study

To validate the content of the survey, experts in pediatric cardiology, cardiothoracic surgery, and public health assessed the clarity, comprehension, and relevance of each question to the measured outcome. Post validation, a pilot study was conducted on 5–10 participants from each university. We employed Cronbach’s alpha to evaluate the reliability and internal consistency of the survey, which was acceptable for knowledge (0.65), attitude (0.67), and practice Sects. (0.7).

### Ethical considerations

The study was conducted according to the principles of the Declaration of Helsinki (1964, last revision 2013). This survey was voluntary and informed consent was obtained from all subjects before completing the questionnaire. Participants’ anonymity and confidentiality were ensured throughout the study**.** Ethical approval was obtained from the Ethical Committee for Scientific Research at the Faculty of Medicine, South Valley University (423, code: 4).

### Statistical analysis

The data were organized in Microsoft Excel and analyzed using IBM SPSS version 27. Frequencies and percentages were used to describe the categorical variables for baseline demographic characteristics. The normality of the continuous variables was assessed using the Shapiro–Wilk test. The median and interquartile range (IQR) were used to describe the non-parametric variables.

Answers to the knowledge questions were coded as 1 for correct and 0 for incorrect or uncertain yielding a score of 0–28 per participant. The description was done by calculating the frequency and percentage for each question as well as the mean and standard deviation for the overall knowledge score. We adopted a cutoff point for each knowledge category based on the score; < 50% of the maximum achievable score to be poor, a score of 50–75%, and > 75% fair and satisfactory, respectively*.*

In the attitude section, the first four questions indicated the perception of neglect while the remaining 11 questions indicated the perception of barriers (Supplementary Table 2). The coding of the answers for questions representing a positive perception of attitude was as follows: strongly disagree = 1, disagree = 2, neutral = 3, agree = 4, and strongly agree = 5. Reverse coding was used for answers to negative questions, resulting in a total score of 4–20 for the perception of neglect and 11–55 for the perception of barriers. The attitude was described using the frequency and percentage for each question as well as the mean and standard deviation for the score for each of the two categories.

The codes for the answers to the practice questions**:** Never = 1, Less likely to = 2, Sometimes = 3, Often = 4, and Always = 5 (positive practices), and reverse coding for answers to negative questions giving a total score of 14–70 (Supplementary Table 2). The practice was described using the frequency and percentage for each question as well as the mean and standard deviation for the overall practice score. The level of participants in overall practice was classified on the maximum achievable score with the same cutoff points as the knowledge section*.*

The chi-square test was used to assess the association between knowledge levels and demographic characteristics. Mann–Whitney and Kruskal–Wallis tests were used to assess the association between demographic characteristics, attitude neglect, barriers, and practice scores using the median and interquartile range. Pearson’s correlation analysis was used to assess the correlation between knowledge, attitude, and practice scores. A *p*-value of ≤ 0.05 was considered significant to test all hypotheses.

## Results

### Demographic data of the participants

**Fig. 2:**
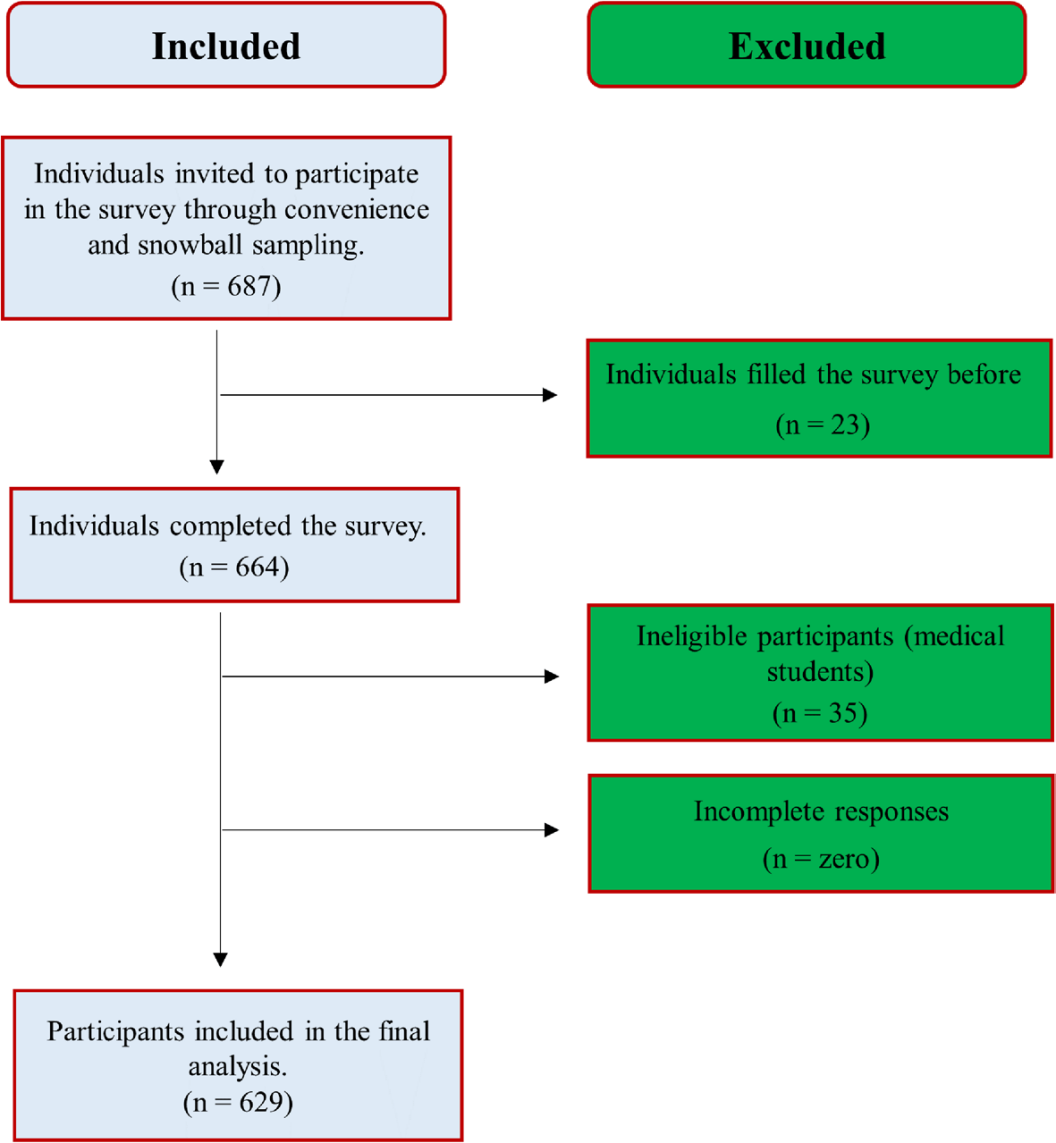
Details of the included and excluded participants

A total of 687 healthcare professionals filled out the questionnaire, of which 629 were included in the final analysis. Included and excluded participants are described in Fig. 2. Male participants were 372, (59.1%). Participants from the Delta region accounted for 53.3% (*n* = 335). Participants were in direct contact with rheumatic fever patients in 40.9% (*n* = 257). Demographic characteristics are described in Table 1.

**Table 1:** Demographic characteristics of the participants

Demographic characteristic	*N* (%) (*N* = 629)
**Gender**	
Male	372 (59.1%)
Female	257 (40.9%)
**University Region**	
Cairo region	117 (18.6%)
Upper Egypt	177 (28.1%)
Delta Region	335 (53.3%)
**Department**	
Departments dealing with RF patients	257 (40.9%)
Departments not dealing with RF patients	372 (59.1%)
**Medical Degree**	
Assistant lecturer physician^a^	150 (23.8%)
House officer and first-year resident	321 (51%)
Second and third-year resident	158 (25.1%)

^a^The assistant lecturer physician is a physician who works as a demonstrator at the university. It is a university job title in Egypt that is earned by obtaining a master’s degree in medicine after residency

### Knowledge about pharyngitis and ARF among physicians

**Fig. 3:**
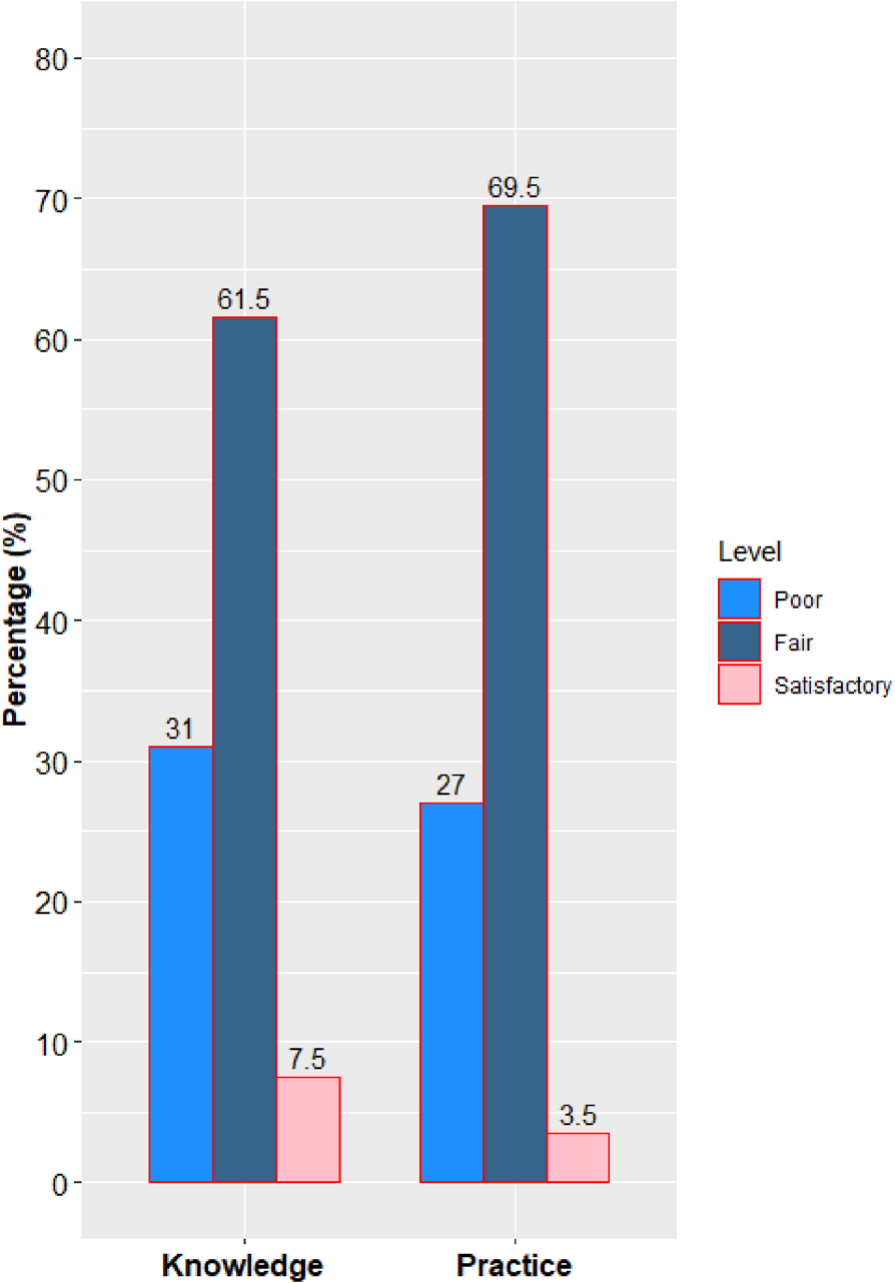
Percentages of knowledge and practice levels among the participants

Participants had a mean overall knowledge score of 17.18 (SD = 3.4) reflecting a generally fair knowledge level. Poor level was detected in 31%, fair in 61.5%, and satisfactory in 7.5% (Fig. 3). Regarding knowledge about pharyngitis, the mean score was 10 (SD = 2.5) reflecting a fair level and only 13.7% had a satisfactory knowledge level. The mean knowledge score for ARF was 7.1 (SD = 1.7) reflecting a fair level, and only 21.6% showed a satisfactory knowledge level. A mean score of 5.5 (SD = 1.5) was obtained in the knowledge questions regarding the etiology while it was 9 (SD = 2) in the knowledge questions regarding the diagnosis. A lower mean score of 2.5 (SD = 1) was detected in the knowledge questions regarding the management of pharyngitis and ARF.

The highest frequency of wrong answers was seen in the use of rapid antigen tests (RAT) for suspected bacterial pharyngitis (77.9%). The highest frequency of wrong answers on the management of ARF and pharyngitis were seen in the alternative to penicillin (75.7%), the duration of antibiotic therapy (70.4%), and the need for antibiotics in the management of ARF in the absence of active bacterial infection (65%).

Regarding the major criteria for ARF diagnosis, the most recognized were polyarthritis (81.2%), endocarditis (70.4%), and subcutaneous nodules (67.4%). The least recognized criteria were valve regurgitation (29.1%), polyarthralgia (21.9%), annular non-pruritic transient rash (21.5%), and monoarthritis (16.4%). The most frequently minor criteria recognized as major were diffuse maculopapular rash (21.6%) and ataxia (20.2%). The participants’ answers to knowledge questions are shown in Supplementary Table 3.

### Attitudes of physicians

The mean score of the perception of neglect was 13.9 (SD = 2.1), while the mean score of the barriers was 36.5 (SD = 4.4), reflecting a high level of neglect and a high burden of barriers towards health promotion.

The most frequently anticipated barriers were the low socioeconomic status of the population (90%), the lack of adequate public education about the disease (85.4%), the widespread public misconceptions (79.5%), and the price of the medications (79.2%). The participants’ answers to knowledge questions are shown in Supplementary Table 4.

### Practices of physicians

Most of the participants (69.5%) had a fair level of practice with a mean of 45.9 (SD = 6.3). Satisfactory levels of practice were in 3.5% (Fig. 3). The highest frequency of poor practices was noticed in the administration of oral antibiotics (63.6%), advising to take a home prescription directly (46.1%), prescribing antibiotics empirically(34.7%), and not seeking medical advice should the participants themselves have a sore throat (28.6%). The participants’ answers to knowledge questions are shown in Supplementary Table 5.

### Associations between the physicians' levels of knowledge, attitude, and practices, and demographics

Physicians who deal directly with ARF patients showed a significantly higher level of satisfactory knowledge about pharyngitis compared to those who have no direct contact with ARF patients (17.1% vs. 11.3%) (*p* = 0.036). Similarly, dealing directly with ARF cases was associated with higher percentages of satisfactory knowledge about ARF (26.8% vs. 18%) (*p* = 0.008). Moreover, physicians in Cairo region universities had a significantly higher percentage of satisfactory knowledge compared to those in Upper Egypt and Delta region universities (31.6% vs. 19.8% and 19.1, respectively) (*p* = 0.014) (Tables 2 and 3).

**Table 2:** Comparison of knowledge of pharyngitis according to demographic data

Demographic characteristic	Poor and fair knowledge (*N* = 543)	Satisfactory knowledge (*N* = 86)	*P*-value*
**Gender**			
- Male	320 (86%)	52 (14%)	0.788
- Female	223 (86.8%)	34 (13.2%)	
**University Region**			
- Cairo region	107 (91.5%)	10 (8.5%)	0.198
- Upper Egypt	150 (84.7%)	27 (15.3%)	
- Delta Region	286 (85.4%)	49 (14.6%)	
**Department**			
- Departments dealing with RF patients	213 (82.9%)	44 (17.1%)	**0.036**
- Departments not dealing with RF patients	330 (88.7%)	42 (11.3%)	
**Medical Degree**			
- Assistant lecturer physician	121 (80.7%)	29 (19.3%)	0.067
- House officer and first-year resident	282 (87.9%)	39 (12.1%)	
- Second and third-year resident	140 (88.6%)	18 (11.4%)	

^*^Chi square test; significant *p*-value < 0.05

**Table 3:** Comparison of knowledge of ARF according to demographic data

**Demographic characteristic**	**Poor and fair knowledge** **(*N* = 493)**	**Satisfactory knowledge** **(*N* = 136)**	*P*-value*
**Gender**			
- Male	294 (79%)	78 (21%)	0.632
- Female	199 (77.4%)	58 (22.6%)	
**University Region**			
- Cairo region	80 (68.4%)	37 (31.6%)	**0.014**
- Upper Egypt	142 (80.2%)	35 (19.8%)	
- Delta Region	271 (80.9%)	64 (19.1%)	
**Department**			
- Departments dealing with RF patients	188 (73.2%)	69 (26.8%)	**0.008**
- Departments not dealing with RF patients	305 (82%)	67 (18%)	
**Medical Degree**			
- Assistant lecturer physician	116 (77.3%)	34 (22.7%)	0.935
- House officer and first-year resident	253 (78.8%)	68 (21.2%)	
- Second and third-year resident	124 (78.5%)	34 (21.5%)	

*Chi square test; significant *p*-value < 0.05

Regarding practices of pharyngitis and ARF, physicians working in Delta region universities had significantly lower levels of positive practices than those working in Upper Egypt or Cairo region universities (*p* = 0.027) (Tables 4 and 5).

**Table 4:** Comparison of attitude and practice scores according to gender and department

		**Gender**		*P*-value*	**Department**		*P*-value*
		**Male**	**Female**		**Departments dealing with RF patients**	**Departments not dealing with RF patients**	
**Attitude**	**Neglect**	14 (3)	14 (4)	0.451	14 (3)	14 (4)	0.102
	**Barriers**	36 (6)	36 (5)	0.860	36 (6)	36 (5)	0.237
**Practice score**		46 (8)	47 (9.5)	0.643	47 (7)	46 (9)	0.130

The data is presented with the median (IQR)

^*^Mann–Whitney test; significant *p*-value < 0.05

**Table 5:** Comparison of attitude and practice scores according to university region and degree

		**University Region**			*P*-value*	**Degree**			*P*-value*
		**Cairo**	**Upper Egypt**	**Delta**		**Assistant physician lecturer**	**House officer, first-year resident**	**Second, third-year resident**	
**Attitude**	**Neglect**	14 (3)	14 (3)	14 (4)	0.33	14 (4)	14 (3)	14 (3)	0.106
	**Barriers**	36 (6)	36 (6)	36 (6)	0.685	36 (5.25)	37 (7)	36 (5)	0.056
**Practice score**		47 (9.5)	47 (8)	45 (9)	**0.027**	46 (8)	46 (8.5)	46 (9)	0.689

The data is presented with the median (IQR)

^*^Kruskal Wallis test; significant *p*-value < 0.05

### Correlations between knowledge, perceptions, and practices scores

We found a significant correlation between the knowledge level of ARF and practice level (*r* = 0.175) *(p-value* < *0.001),* but no significant correlation between the knowledge level of pharyngitis and practice levels. A weak positive correlation was found between the knowledge level and the perception of of barriers (*r* = 0.177) (*p-value* < *0.001)*. Moreover, there was a weak negative correlation between the practice level and the perception of barriers (*r* =—0.162) *(p-value* < *0.001)*.

## Discussion

RHD remains a significant problem in Egypt that is preventable by adequate management of bacterial pharyngitis and ARF [1]. This study revealed a fair level of knowledge and practices regarding bacterial pharyngitis and ARF among frontline university physicians. Nevertheless, it identified many gaps contributing to the mismanagement of RHD in Egypt.

### Overall knowledge of physicians

Although only 7.5% of physicians had satisfactory knowledge, around 69% scored > 50% which is higher than the results of a study performed in Addis Ababa including nursing staff where 48.7% scored above 50% [15]. A study including senior medical students in Cameroon showed better knowledge, maybe because the students had been recently educated about pharyngitis and ARF [14]. Our study showed better knowledge about etiology and diagnosis compared to the management of pharyngitis and ARF explaining in part the higher prevalence in Egypt [9]. Although most participants had fair knowledge levels, those dealing directly with ARF cases had significantly better knowledge compared to those who did not. Noteworthy, primary health care and family medicine in Egypt remain unfamiliar to the public. Sore throat patients sporadically consult primary, secondary, or tertiary care physicians in outpatient clinics, university hospitals, and the private sector either conveniently or based on factors like counseling price and location rather than the department or specialty [16]. It can be argued that the defect in the knowledge or practice of physicians contributes to the burden of ARF and RHD.

### Knowledge about pharyngitis

Only 7.6% of participants knew all the Centor criteria of bacterial pharyngitis. In a Sudanese study, 3% of the house officers identified all criteria [17]. To diagnose bacterial pharyngitis, 22.1% selected RAT and 39.4% chose throat culture (TC). Although TC is the gold standard diagnostic test, guidelines recommend the RAT initially for its high specificity, while only if the RAT is negative, TC should be performed to rule out the disease [18]. The lack of local availability of the RAT may contribute to this result. This divergence in answers appeared again in the management where more than half reported that antibiotics should be administered for 5–7 days while only 29.6% chose to continue for 10 days, which is currently recommended by the guidelines given the significant relation between antibiotic duration and prevention of ARF [18]. Although the majority identified penicillin as the first-line antibiotic, the responses diverged again on the alternatives, where 51.7% selected azithromycin and 24.3% chose cephalosporins. Except for an anaphylactic allergy, cephalosporins are increasingly recommended as alternatives to penicillin due to their high efficacy and the increasing resistance and weaker evidence for macrolides [18].

### Knowledge about ARF

Less than 1% identified all Major Jones Criteria of ARF**,** and 21.9% and 16.4% identifed polyarthralgia and monoarthritis as major criteria. In their revision of the Jones Criteria, Gewitz et al. mentioned that both polyarthralgia and monoarthritis increased the sensitivity of ARF diagnosis when they were considered major criteria in high-risk populations [11, 19]. Our findings explain in part the high rate of misdiagnosis found by Ghamrawy et al. [9]. Possibly, Egyptian physicians mainly rely on clinical experience rather than guidelines. The use of antibiotics for ARF in the absence of active GAS infection was known to 35% of participants, this is a cornerstone for secondary prevention of RHD [12].

The results of the knowledge section raise concerns regarding the clinical practice of Egyptian physicians. It is unclear whether these gaps in knowledge appear only in clinical practice after graduation or result from a defect in the educational system for medical students in the first place.

### The attitude of physicians towards pharyngitis and ARF

Most respondents perceived ARF to be neglected with patient negligence of sore throat being the highest (79%) followed by general underreporting (65.8%). In addition, a high burden of contributing barriers was perceived with the socioeconomic status being the highest (90%) [20]. The lack of public education and the widespread public misconceptions about the disease came second. Interestingly, the price of medication and its availability came third; suggesting that working on patient-specific factors like economic status and education might be more impactful compared to medication-related factors.

### Practices of physicians regarding pharyngitis and ARF

Less than half of physicians seek medical advice if they have a sore throat. This is less than the 69.3% found by a study on 6958 Egyptians [2]. This could be explained by overconfidence and self-medication of healthcare practitioners compared to the general population [21]. Although 38.4% would not prescribe antibiotics for sore throat before confirming its bacterial origin, the knowledge about using the Centor criteria and RAT or TC was disappointing. Also, 34.7% say they prescribe empiric antibiotics before confirming the diagnosis, while 46.1% give a home prescription without examining the patient. Unnecessary prescription of antibiotics contributes fiercely to the emerging problem of antimicrobial resistance [22, 23]. The decision-making process for management in clinical practice in Egypt needs to be extensively updated, unified and tracked.

### The use of echocardiography for ARF

While more than 65% of the respondents recommend echocardiography in confirmed ARF even without clinical carditis, 48% recommend it for suspected unconfirmed cases. It has been debated that echocardiography can diagnose subtle “subclinical” carditis [17, 24]. Several studies found an increased sensitivity in diagnosing ARF when subclinical carditis is considered a major criterion [19, 25]. Therefore, the WHO recommends its use in confirmed ARF regardless of clinical carditis [11, 26]. Whether transthoracic or transesophageal echocardiography should be performed, and how often lies beyond the scope of this study.

### Knowledge and practice across the geographic regions

The current study indicated no significant differences in knowledge of pharyngitis among the three regions. However, as expected, the Cairo region had significantly higher levels of satisfactory knowledge about ARF compared to the Delta and Upper Egypt regions. Furthermore, the Delta region showed significantly lower positive practice levels compared to the other two regions. This indicates that practice is better in the Upper Egypt region compared to the Delta region despite the lower level of knowledge. This discrepancy might be explained by exposure to patients. It can be argued that the estimated prevalence and, subsequently, the exposure to ARF patients in Upper Egypt is higher than in the Delta regions due to the lower socioeconomic status. [20]. This might have increased awareness of Upper Egypt physicians resulting in better practices. The knowledge section of the survey is based on knowledge about the updated guidelines while the practice section relied on general medical practices.

### Correlations between knowledge, attitudes, and practices

A poor positive correlation was found between knowledge and perception of barriers reflecting an increased consciousness about how barriers might contribute to the problem as knowledge increases. Moreover, practices correlated negatively with the perception of barriers. Finally, a linear correlation was noted between knowledge about ARF and practices which is in line with the positive correlation found in a Sudanese study among house officers [27]. No significant correlation was detected between knowledge of pharyngitis and practices. Thus, targeting the knowledge about ARF rather than pharyngitis can potentially improve overall practices.

### Strengths

To our knowledge, this is the first multicenter, cross-sectional study investigating the primary and secondary prevention of RHD through the healthcare system in Egypt. Our study targeted the frontline healthcare providers in university hospitals. It has highlighted important gaps in knowledge and practice that could help implement targeted interventions in concordance with the 2006 National RHD Prevention and Control Program in Egypt. The study also provides a statistically validated survey to be used in future research. The larger sample size increased the generalizability, and the anonymous distribution reduced the response bias.

### Limitations

Despite the strengths, our study had some limitations. A limitation of the study lies in the convenience and snowball sampling methods, which may result in selection bias. It yielded a larger sample from the Delta region compared to the other two regions. This may have impacted the observations and therefore interfered with generalizability. However, it might also indicate the magnitude of the rheumatic heart disease problem and the awareness in this region. Also, the survey was too long according to some participants. Finally, we did not include primary care and family physicians in this survey due to their limited representation in Egyptian university hospitals.

### Recommendations

Based on the study results, we recommend targeted education of all healthcare departments in Egyptian universities with an emphasis on proper management. This could be followed by pre- and post-intervention reassessment studies [28]. Also, raising public awareness must improve early diagnosis and combat the high levels of neglect. It is important to update the local guidelines in Egypt for screening, diagnosis, and management of bacterial pharyngitis, ARF, and RHD based on the most recent international guidelines [9, 11–13]. The updated guidelines should then be implemented within the National RHD Prevention and Control Program in Egypt followed by an assessment of adherence [29]. Additionally, a clear role and responsibility of primary health care and family medicine should be stated in the health care system with strict referral and admission processes for patients. This way they can play a crucial role in reducing the magnitude of ARF and RHD. Furthermore, it is important to investigate the impact of the medical educational system in Egypt on the magnitude of the problem which can represent an important aspect of the equation. Future research on prevalence, morbidity, mortality, and health care utilization and its financial burden regarding ARF and RHD is recommended.

## Conclusions

A cross-sectional survey was conducted among Egyptian frontline physicians in different universities on the knowledge, attitudes, and practices regarding bacterial pharyngitis and ARF. The study yielded fair knowledge and practice levels. However, we identified many potential gaps based upon which we recommended targeted educational interventions to be implemented by updating the local guidelines in Egypt for diagnosis and management based on the most recent guidelines. Further extensive research on the impact of the healthcare sector could help us tackle the problem of RHD in high-prevalence countries.

### Supplementary Information

Supplementary Material 1.

## Data Availability

The data generated and analyzed are shown in the manuscript and supplementary files. Further inquiries should be directed to the corresponding author.

## Abbreviations

RHD: Rheumatic heart disease
ARF: Acute rheumatic fever
GAS: Group A Streptococcal
WHO: World Health Organization
STROBE: Strengthening The Reporting of Observational Studies in Epidemiology
IQR: Interquartile range
SD: Standard deviation
RAT: Rapid antigen test
TC: Throat culture
