# Supplementary material for: Assessing the knowledge, attitude, and practice of frontline physicians in Egyptian university hospitals regarding pharyngitis and acute rheumatic fever: a cross-sectional study that calls for action
Source: BMC Public Health. 2024 Aug 16;24:2235. doi: 10.1186/s12889-024-19658-5 (PMC11330028; doi:10.1186/s12889-024-19658-5)
Supplement: Supplementary file 1 — Supplementary Material 1. [file 12889_2024_19658_MOESM1_ESM.pdf]

Supplementary Table 1. List of the included universities in the study.

Universities
• Cairo University<br>• Ain Shams University<br>• Al Azhar Cairo University<br>• Helwan University<br>• Minya University<br>• Al Azhar Assiut University<br>• Assiut University<br>• Sohag University<br>• South Valley University<br>• Aswan University<br>• Alexandria University<br>• Benha University<br>• Tanta University<br>• Al Azhar Damietta University<br>• Mansoura University<br>• Menofia University<br>• Suez Canal University<br>• Kafr El Sheikh University<br>• Zagazig University<br>• Fayoum University<br>• Port said University

Supplementary Table 2. Knowledge, attitude, and practice questionnaire

Knowledge Domain (Correct answers are **underlined in Bold**)

1- Pharyngitis

1] Etiology

1. **What contributes to pharyngitis more frequently?**
   Bacteria – **Viral** – Both contribute equally – I don't know.

2. **Which of the following is the most common cause of bacterial pharyngitis?**
   GAS – Pneumococci – Staph aureus – Neisseria meningitidis – Haemophiles influenzae – I don't know.

3. **What is the most common method by which bacterial pharyngitis is transmitted?**
   Droplets – Aerosol – Direct contact with skin lesions – All of them – I don't know.

4. **What is the most common age group affected with bacterial pharyngitis?**
   (<5) – (**5 to 15**) – (16 to 44) – (>44) – I don't know.

2] Diagnosis

5. **Does the presence of rhinorrhea more commonly suggest bacterial or viral pharyngitis?**
   Bacterial – **Viral** – Both equally – I don't know.

6. **Does the presence of tonsillitis more commonly suggest bacterial or viral pharyngitis?**
   Bacterial – **Viral** – Both equally – I don't know.

7. **Does the presence of conjunctivitis more commonly suggest bacterial or viral pharyngitis?**
   Bacterial – Viral – Both equally – I don't know.

8. **Does the presence of cough more commonly suggest bacterial or viral pharyngitis?**
   Bacterial – **Viral** – Both equally – I don't know.

9. **Does the presence of exudate with pharyngitis more commonly suggest bacterial or viral pharyngitis?**
   Bacterial – Viral – Both equally – I don't know.

10. **Do patients with bacterial pharyngitis most commonly present with fever?**
    Yes – No – Maybe or maybe not – I don't know.

11. **Do patients with bacterial pharyngitis most commonly present with cervical lymphadenopathy?**
    Yes – No – **Maybe or maybe not** – I don't know.

12. **Which technique is recommended initially for the diagnosis of clinically suspected bacterial pharyngitis?**
    Rapid antigen test – Throat culture – Gram stain – Bacterial anti-body titer – Antibiotic testing – PCR – I don't know.

13. **If the initial diagnostic test for suspected bacterial pharyngitis was negative in a child with pharyngitis, what is the next step in management?**
    Confirm by another test – Start antibiotics – Conservative management – I don't know.

14. **If the initial test for suspected bacterial pharyngitis was positive in a child with pharyngitis, what is the next step in management?**
    Confirm by another test – **Start antibiotics** – Conservative management – I don't know.

3] Management

15. **What is the drug of choice for the treatment of bacterial pharyngitis?**
    Penicillin – Cephalosporins – Azithromycin – Clindamycin – I don't know.

16. **What is the drug of choice for the treatment of allergic patients to first-line treatment?**
    Penicillin – **Cephalosporins** – Azithromycin – Clindamycin – I don't know.

17. **What is the recommended duration of antibiotic therapy for bacterial pharyngitis?**
    3 days – 5 days – 7 days – **10 days** – I don't know.

2- Acute rheumatic fever

1] Etiology

1. **What is the primary bacterial infection that led eventually to acute rheumatic fever?**
   Pharyngitis – Skin infection – Both can contribute – Neither of them contributes – I don't know.

2. **When does acute rheumatic fever usually develop following the primary infection?**
   (<1) week – **(1-5) weeks** – (6-12) weeks – (3-6) months – (6-12) months – (>1) year – I don't know.

3. **Which age group is most affected by acute rheumatic fever?**
   (<3) – **(3 to 14)** – (15 to 44) – (>44) – I don't know.

4. **Can acute rheumatic fever turn into a chronic disease?**
   Yes – No – I don't know.

2] Diagnosis

5. **Which of the following are considered major symptoms for diagnosing acute rheumatic fever? (CHECK-BOXES)**
   - **Polyarthritis**
   - Polyarthralgia
   - Monoarthritis
   - Monoarthralgia

   - **Pericarditis**
   - **Myocarditis**
   - **Endocarditis**
   - **Valve regurgitation**
   - Valve stenosis

   - **Subcutaneous nodules**
   - Bilateral painful shin (knee) nodules
   - **Annular non-pruritic transient rash**

- Diffuse maculopapular rash
- **Jerky involuntary movements of limbs**
- Ataxia

6. **Is the diagnosis of acute rheumatic fever supported by increased ESR/CRP?**
   Yes – No – I don't know

7. **Is the diagnosis of acute rheumatic fever supported by the presence of fever?**
   Yes – No – I don't know

8. **Is laboratory evidence of previous bacterial infection required for a diagnosis of acute rheumatic fever?**
   Yes – No – I don't know

9. **In evidence of previous bacterial infection, how many criteria (major or minor) should be present for the diagnosis of acute rheumatic fever?**
   - (1 major alone) or (2 minors alone)
   - (**2 majors alone) or (1 major + 2 minors)**
   - (3 majors alone) or (2 majors + 1 minor)
   - (1 major + 1 minor) or (3 minors alone)
   - I don't know.

3] Management

10. **Is acute rheumatic fever preventable by adequate antibiotic therapy for the primary bacterial infection?**
    Yes – No – I don't know.

11. **Should antibiotics be used during the management of acute rheumatic fever in the absence of active bacterial infection?**
    Always – Only in certain cases – No – I don't know.

Attitude Domain

Answer choices for all questions are: Strongly agree – Agree – Not sure – Disagree – Strongly disagree.

Questions with (R): reverse coding

1- Perception of neglect

1. **Sore throat is usually neglected by patients in Egypt.**
2. **Patients in Egypt with bacterial pharyngitis would complete the full course of antibiotics recommended by their primary healthcare physician. (R)**
3. **Acute rheumatic fever and rheumatic heart diseases are underreported in Egypt. (R)**
4. **Acute rheumatic fever is diagnosed early in Egypt.**

2- Perception of barriers

5. **Teaching about acute rheumatic fever and rheumatic heart disease in Egyptian medical schools is sufficient and actively contributes to decreasing the prevalence of the disease.**
6. **Low socioeconomic status is a risk factor for the development of acute rheumatic fever. (R)**

7. **Current guidelines are adequate for accurate diagnosis of pharyngitis. (R)**
8. **Current guidelines are adequate for accurate diagnosis of acute rheumatic fever. (R)**
9. **Current research on acute rheumatic fever/rheumatic heart disease in Egypt is satisfactory and actively eradicating the problem. (R)**
10. **Specialists' contribution to public health awareness about rheumatic fever/rheumatic heart disease in Egypt is satisfactory and actively eradicates the problem. (R)**
11. **Government funding for public health awareness about rheumatic fever/rheumatic heart disease in Egypt is satisfactory and actively eradicates the problem. (R)**
12. **Price is an important contributing barrier to patients' compliance with acute pharyngitis/acute rheumatic fever treatments.**
13. **Availability of medications is an important contributing barrier to patients' compliance with acute pharyngitis/acute rheumatic fever treatments.**
14. **Cultural misconceptions about the disease or treatment's importance are important contributing barriers to patients' compliance with acute pharyngitis/acute rheumatic fever treatments.**
15. **Lack of adequate education about the disease or treatment importance is an important contributing barrier to patients' compliance with acute pharyngitis/acute rheumatic fever treatments.**

Practice Domain

Answer choices for all questions are: Always – Often – Sometimes – Less likely to – Never.

Questions with (R): reverse coding

1. **Would you seek medical advice if you had an episode of a sore throat?**
2. **Would you ask anyone with a sore throat to visit a doctor?**
3. **Would you ask a patient with a sore throat to take a home prescription directly? (R)**
4. **Would you prescribe empiric antibiotics to a patient with pharyngitis before confirming it is of bacterial origin? (R)**
5. **Would you recommend Echocardiography with Doppler for all cases of suspected acute rheumatic fever before confirmation?**
6. **Would you recommend Echocardiography with Doppler for all cases of confirmed acute rheumatic fever even with no evidence of carditis?**
7. **How likely are you to admit a patient with suspected acute rheumatic fever to the hospital?**
8. **How often do you educate your patients about rheumatic fever and its complications?**
9. **How often do you educate your patients about the importance of home measures like self-hygiene in the prevention of sore throat?**
10. **Regarding antibiotic administration, how likely are you to administer antibiotics orally? (R)**
11. **Regarding antibiotic administration, how likely are you to administer antibiotics parenterally?**
12. **How likely are you to follow up with your patients until you make sure they complete their antibiotic course?**
13. **How likely are you to advocate for the establishment of a rheumatic heart disease registry in Egypt?**
14. **How likely are you to participate in a research project on acute rheumatic fever/rheumatic heart disease?**

Supplementary Table 3. Responses of the participants to the knowledge questions

**Knowledge about pharyngitis**	**N (%) (N= 629)**
**1- What contributes to pharyngitis more frequently?**	
o Bacteria	213 (33.9%)
o Viral	342 (54.4%)
o Both contribute equally.	64 (10.2%)
o I don't know.	10 (1.5%)
**2- Which of the following is the most common cause of bacterial pharyngitis?**	
o Group A streptococcus	466 (74.1%)
o Staph aureus	84 (13.4%)
o Streptococcus pneumonia	42 (6.7 %)
o Haemophiles influenzae	11 (1.7 %)
o I don't know.	26 (4.1%)
**3- What is the most common method by which bacterial pharyngitis is transmitted?**	
o Droplets	440 (70%)
o Aerosol	45 (7.2%)
o Direct contact with skin lesions	13 (2.1%)
o All of them	116 (18.4%)
o I don't know.	15 (2.3%)
**4- What is the most common age group affected with bacterial pharyngitis?**	
o <5	127 (20.2%)
o 5-15	444 (70.6%)
o 16-44	20 (3.2%)
o >44	3 (0.5%)
o I don't know.	35 (5.5%)
**5- Does the presence of rhinorrhea more commonly suggest bacterial or viral pharyngitis?**	
o Bacterial	29 (4.6%)
o Viral	508 (80.8%)
o Both equally	67 (10.7%)
o I don't know.	25 (3.9%)
**6- Does the presence of tonsillitis more commonly suggest bacterial or viral pharyngitis?**	
o Bacterial	420 (66.8%)

○ Viral<br>○ Both equally<br>○ I don't know.	87 (13.8%)<br>104 (16.5%)<br>18 (2.9%)
**7- Does the presence of conjunctivitis more commonly suggest bacterial or viral pharyngitis?**<br>○ Bacterial<br>○ Viral<br>○ Both equally<br>○ I don't know.	74 (11.8%)<br>430 (68.4%)<br>65 (10.3%)<br>60 (9.5%)
**8- Does the presence of cough more commonly suggest bacterial or viral pharyngitis?**<br>○ Bacterial<br>○ Viral<br>○ Both equally<br>○ I don't know.	116 (18.4%)<br>252 (40.1%)<br>219 (34.8%)<br>42 (6.7%)
**9- Does the presence of exudate with pharyngitis more commonly suggest bacterial or viral pharyngitis?**<br>○ Bacterial<br>○ Viral<br>○ Both equally<br>○ I don't know.	517 (82.2%)<br>42 (6.7%)<br>61 (9.7%)<br>9 (1.4%)
**10- Do patients with bacterial pharyngitis most commonly present with fever?**<br>○ Yes<br>○ No<br>○ Maybe or maybe not<br>○ I don't know.	490 (77.9%)<br>15 (2.4%)<br>109 (17.3%)<br>15 (2.4%)
**11- Do patients with bacterial pharyngitis most commonly present with cervical lymphadenopathy?**<br>○ Yes<br>○ No<br>○ Maybe or maybe not<br>○ I don't know.	298 (47.4%)<br>55 (8.7%)<br>246 (39.1%)<br>30 (4.8%)
**12- Which diagnostic test is recommended initially for diagnosis of a clinically suspected bacterial pharyngitis?**<br>○ Rapid antigen test<br>○ Throat culture	139 (22.1%)<br>248 (39.4%)<br>49 (7.8%)

○ Gram stain<br>○ Bacterial anti-body titer<br>○ Antibiotic testing<br>○ PCR<br>○ I don't know.	79 (12.6%)<br>30 (4.8%)<br>8 (1.3%)<br>76 (12%)
**13- If the initial diagnostic test for a suspected bacterial pharyngitis was negative in a child with pharyngitis, what is the next step in management?**<br>○ Confirm by another test.<br>○ Start antibiotics.<br>○ Conservative management<br>○ I don't know.	 211 (33.5%)<br>79 (12.6%)<br>298 (47.4%)<br>41 (6.5%)
**14- If the initial test for a suspected bacterial pharyngitis was positive in a child with pharyngitis, what is the next step in management?**<br>○ Confirm by another test.<br>○ Start antibiotics.<br>○ Conservative management<br>○ I don't know.	 43 (6.8%)<br>547 (87%)<br>19 (3%)<br>20 (3.2%)
**15- What is the drug of choice for the treatment of bacterial pharyngitis?**<br>○ Penicillin<br>○ Cephalosporins<br>○ Azithromycin<br>○ Clindamycin<br>○ I don't know.	 527 (83.8%)<br>49 (7.8%)<br>23 (3.7%)<br>7 (1.1%)<br>23 (3.6%)
**16- What is the drug of choice for the treatment of allergic patients to first line treatment?**<br>○ Penicillin<br>○ Cephalosporins<br>○ Azithromycin<br>○ Clindamycin<br>○ I don't know.	 27 (4.3%)<br>153 (24.3%)<br>325 (51.7%)<br>68 (10.8%)<br>56 (8.9%)
**17- What is the recommended duration of antibiotic therapy for bacterial pharyngitis?**<br>○ 1-3 days<br>○ 3-5 days<br>○ 5-7 days<br>○ 10 days<br>○ I don't know.	 12 (1.9%)<br>67 (10.7%)<br>341 (54.2%)<br>186 (29.6%)<br>23 (3.6%)

Knowledge about ARF	N (%) (N= 629)
**1- What is the primary bacterial infection that led eventually to acute rheumatic fever?**	
o Pharyngitis	396 (63%)
o Skin infection	10 (1.6%)
o Both can contribute.	198 (31.5%)
o Neither of them contributes	9 (1.4%)
o I don't know.	16 (2.5%)
**2- When does acute rheumatic fever usually develop following the primary infection?**	
o (<1) week	25 (4%)
o (1-5) weeks	339 (53.9%)
o (6-12) weeks	91 (14.5%)
o (3-6) months	32 (5.1%)
o (6-12) months	19 (3%)
o (>1) year	10 (1.6%)
o I don't know.	113 (17.9%)
**3- Which age group is most affected by acute rheumatic fever?**	
o (<3)	28 (4.5%)
o (3-14)	513 (81.5%)
o (15-44)	63 (10%)
o (>44)	3 (0.5%)
o I don't know.	22 (3.5%)
**4- Can acute rheumatic fever turn into a chronic disease?**	
o Yes	564 (89.7%)
o No	28 (4.5%)
o I don't know.	37 (5.8%)
**5- Which of the following symptoms are considered major symptoms for the diagnosis of acute rheumatic fever? (CHECK-BOX)**	
o Polyarthritis	511 (81.2%)
o Polyarthralgia	138 (21.9%)
o Monoarthritis	103 (16.4%)
o Monarthralgia	16 (2.5%)
o Pericarditis	355 (56.4%)
o Myocarditis	394 (62.6%)
o Endocarditis	443 (70.4%)

o Valve regurgitation	183 (29.1%)
o Valve stenosis	114 (18.1%)
o Subcutaneous nodules	424 (67.4%)
o Bilateral painful shin (knee) nodules	116 (18.4%)
o Annular non-pruritic transient rash	135 (21.5%)
o Diffuse maculopapular rash.	136 (21.6%)
o Jerky involuntary movements of limbs	301 (47.9%)
o Ataxia	127 (20.2%)
**6- Is diagnosis of acute rheumatic fever supported by increased ESR/CRP?**	
o Yes	558 (88.7%)
o no	49 (7.8%)
o I don't know.	22 (3.5%)
**7- Is diagnosis of acute rheumatic fever supported by the presence of fever?**	
o Yes	496 (78.9%)
o No	96 (15.3%)
o I don't know.	37 (5.8%)
**8- Is laboratory evidence of previous bacterial infection required for a diagnosis of acute rheumatic fever?**	
o Yes	419 (66.6%)
o No	140 (22.3%)
o I don't know.	70 (11.1%)
**9- In evidence of previous bacterial infection, how many criteria (major or minor) should be present for diagnosis of acute rheumatic fever?**	
o (1 major alone) or (2 minors alone)	72 (11.4%)
o (2 majors alone) or (1 major + 2 minors)	448 (71.2%)
o (3 majors alone) or (2 majors + 1 minor)	43 (6.8%)
o (1 major + 1 minor) or (3 minors alone)	35 (5.6%)
o I don't know.	31 (5%)
**10- Is acute rheumatic fever preventable by adequate antibiotic therapy to the primary bacterial infection?**	
o Yes	536 (85.2%)
o No	56 (8.9%)
o I don't know.	37 (5.9%)

**11- Should antibiotics be used during the management of acute rheumatic fever in the absence of active bacterial infection?**<br>o Always<br>o Only in certain cases<br>o No<br>o I don't know.	 220 (35%)<br>227 (36.1%)<br>95 (15.1%)<br>87 (13.8%)

Supplementary Table 4. Participants' responses to the attitude questions (N= 629)

**1- Sore throat is usually neglected by patients in Egypt.**	
o Strongly agree.	190 (30.2%)
o Agree	306 (48.7%)
o Not sure	44 (7%)
o Disagree	78 (12.4%)
o Strongly disagree.	11 (1.7%)
**2- Patients in Egypt with bacterial pharyngitis would complete the full course of antibiotics recommended by their primary health care physician.**	
o Strongly agree.	105 (16.7%)
o Agree	124 (19.7%)
o Not sure	143 (22.7%)
o Disagree	183 (29.1%)
o Strongly disagree.	74 (11.8%)
**3- Acute rheumatic fever is diagnosed early in Egypt.**	
o Strongly agree.	20 (3.2%)
o Agree	103 (16.4%)
o Not sure	212 (33.7%)
o Disagree	260 (41.3%)
o Strongly disagree.	34 (5.4%)
**4- Acute rheumatic fever and rheumatic heart diseases are underreported in Egypt.**	
o Strongly agree.	125 (19.9%)
o Agree	289 (45.9%)
o Not sure	149 (23.7%)
o Disagree	57 (9.1%)
o Strongly disagree.	9 (1.4%)
**5- Low socioeconomic status is a risk factor for the development of acute rheumatic fever.**	
o Strongly agree.	278 (44.2%)
o Agree	288 (45.8%)
o Not sure	50 (7.9%)
o Disagree	12 (1.9%)
o Strongly disagree.	1 (0.2%)
**6- Teaching about acute rheumatic fever and rheumatic heard disease in Egyptian medical schools is sufficient and actively contributing to decrease the prevalence of the disease.**	

○ Strongly agree.<br>○ Agree<br>○ Not sure<br>○ Disagree<br>○ Strongly disagree.	95 (15.1%)<br>242 (38.5%)<br>139 (22.1%)<br>136 (21.6%)<br>17 (2.7%)
7- **Current guidelines are adequate for accurate diagnosis of pharyngitis.**<br>○ Strongly agree.<br>○ Agree<br>○ Not sure<br>○ Disagree<br>○ Strongly disagree.	 66 (10.5%)<br>304 (48.3%)<br>185 (29.4%)<br>58 (9.2%)<br>16 (2.6%)
8- **Current guidelines are adequate for accurate diagnosis of acute rheumatic fever.**<br>○ Strongly agree.<br>○ Agree<br>○ Not sure<br>○ Disagree<br>○ Strongly disagree.	 77 (12.2%)<br>329 (52.3%)<br>169 (26.9%)<br>43 (6.8%)<br>11 (1.8%)
9- **Current research on acute rheumatic fever/rheumatic heart disease in Egypt is satisfactory and actively eradicating the problem.**<br>○ Strongly agree.<br>○ Agree<br>○ Not sure<br>○ Disagree<br>○ Strongly disagree.	 36 (5.7%)<br>162 (25.8%)<br>272 (43.2%)<br>140 (22.3%)<br>19 (3%)
10- **Specialists' contribution to public health awareness about rheumatic fever/rheumatic heart disease in Egypt is satisfactory and actively eradicating the problem.**<br>○ Strongly agree.<br>○ Agree<br>○ Not sure<br>○ Disagree<br>○ Strongly disagree.	 35 (5.6%)<br>196 (31.2%)<br>226 (35.9%)<br>151 (24%)<br>21 (3.3%)
11- **Government funding for public health awareness about rheumatic fever/rheumatic heart disease in Egypt is satisfactory and actively eradicating the problem.**	

○ Strongly agree.<br>○ Agree<br>○ Not sure<br>○ Disagree<br>○ Strongly disagree.	36 (5.7%)<br>141 (22.4%)<br>220 (35%)<br>185 (29.4%)<br>47 (7.5%)
12- **Price is an important contributing barrier to patients' compliance with acute pharyngitis/acute rheumatic fever treatments.**<br>○ Strongly agree.<br>○ Agree<br>○ Not sure<br>○ Disagree<br>○ Strongly disagree.	 168 (26.7%)<br>330 (52.5%)<br>89 (14.1%)<br>35 (5.6%)<br>7 (1.1%)
13- **Availability of medications is an important contributing barrier to patients' compliance with acute pharyngitis/acute rheumatic fever treatments.**<br>○ Strongly agree.<br>○ Agree<br>○ Not sure<br>○ Disagree<br>○ Strongly disagree.	 145 (23%)<br>319 (50.7%)<br>88 (14%)<br>69 (11%)<br>8 (1.3%)
14- **Cultural misconceptions about the disease or treatment importance are important contributing barriers to patients' compliance with acute pharyngitis/acute rheumatic fever treatments.**<br>○ Strongly agree.<br>○ Agree<br>○ Not sure<br>○ Disagree<br>○ Strongly disagree.	 151 (24%)<br>349 (55.5%)<br>109 (17.3%)<br>17 (2.7%)<br>3 (0.5%)
15- **Lack of adequate education about the disease or treatment importance is an important contributing barrier to patients' compliance with acute pharyngitis/acute rheumatic fever treatments.**<br>○ Strongly agree.<br>○ Agree<br>○ Not sure<br>○ Disagree<br>○ Strongly disagree.	 194 (30.9%)<br>343 (54.5%)<br>76 (12.1%)<br>14 (2.2%)<br>2 (0.3%)

Supplementary Table 5. Participants' responses to the practice questions (N= 629)

**1- Would you seek medical advice if you had an episode of sore throat?**	
○ Always	89 (14.2%)
○ Often	178 (28.3%)
○ Sometimes	182 (28.9%)
○ Less likely to	143 (22.7%)
○ Never.	37 (5.9%)
**2- Would you ask anyone with a sore throat to visit a doctor?**	
○ Always	137 (21.8%)
○ Often	214 (34%)
○ Sometimes	171 (27.2%)
○ Less likely to	96 (15.3%)
○ Never.	11 (1.7%)
**3- Would you ask a patient with a sore throat to take a home prescription directly?**	
○ Always	82 (13%)
○ Often	208 (33.1%)
○ Sometimes	200 (31.8%)
○ Less likely to	93 (14.8%)
○ Never.	46 (7.3%)
**4- Would you prescribe empiric antibiotics to a patient with pharyngitis before confirming it is of bacterial origin?**	
○ Always	56 (8.9%)
○ Often	162 (25.8%)
○ Sometimes	169 (26.9%)
○ Less likely to	126 (20%)
○ Never.	116 (18.4%)
**5- Would you recommend Echocardiography with Doppler for all cases of suspected acute rheumatic fever before confirmation?**	
○ Always	121 (19.2%)
○ Often	181 (28.8%)
○ Sometimes	138 (21.9%)

○ Less likely to<br>○ Never.	144 (22.9%)<br>45 (7.2%)
6- **Would you recommend Echocardiography with Doppler for all cases of confirmed acute rheumatic fever even with no evidence of carditis?**<br>○ Always<br>○ Often<br>○ Sometimes<br>○ Less likely to<br>○ Never.	 221 (35.1%)<br>197 (31.3%)<br>111 (17.7%)<br>77 (12.2%)<br>23 (3.7%)
7- **How likely are you to admit a patient with suspected acute rheumatic fever to the hospital?**<br>○ Always<br>○ Often<br>○ Sometimes<br>○ Less likely to<br>○ Never	 113 (18%)<br>190 (30.2%)<br>198 (31.5%)<br>94 (14.9%)<br>34 (5.4%)
8- **How often do you educate your patients about rheumatic fever and its complications?**<br>○ Always<br>○ Often<br>○ Sometimes<br>○ Less likely to<br>○ Never.	 160 (25.4%)<br>215 (34.2%)<br>179 (28.4%)<br>57 (9.1%)<br>18 (2.9%)
9- **How often do you educate your patients about the importance of home measures like self-hygiene in the prevention of sore throat?**<br>○ Always<br>○ Often<br>○ Sometimes<br>○ Less likely to<br>○ Never.	 206 (32.8%)<br>222 (35.3%)<br>143 (22.7%)<br>42 (6.7%)<br>16 (2.5%)
10- **Regarding antibiotic administration, how likely are you to administer antibiotics orally?**<br>○ Always<br>○ Often<br>○ Sometimes	 115 (18.3%)<br>285 (45.3%)<br>178 (28.3%)

○ Less likely to<br>○ Never.	40 (6.4%)<br>11 (1.7%)
11- **Regarding antibiotic administration, how likely are you to administer antibiotics parenteral?**<br>○ Always<br>○ Often<br>○ Sometimes<br>○ Less likely to<br>○ Never.	 48 (7.6%)<br>146 (23.2%)<br>243 (38.6%)<br>164 (26.1%)<br>28 (4.5%)
12- **How likely are you to follow up with your patients until you make sure they completed their antibiotic course?**<br>○ Always<br>○ Often<br>○ Sometimes<br>○ Less likely to<br>○ Never.	 111 (17.6%)<br>211 (33.5%)<br>182 (28.9%)<br>101 (16.2%)<br>24 (3.8%)
13- **How likely are you to advocate for the establishment of a rheumatic heart disease registry in Egypt?**<br>○ Always<br>○ Often<br>○ Sometimes<br>○ Less likely to<br>○ Never.	 111 (17.6%)<br>193 (30.7%)<br>213 (33.9%)<br>82 (13%)<br>30 (4.8%)
14- **How likely are you to participate in a research project on acute rheumatic fever/rheumatic heart disease?**<br>○ Always<br>○ Often<br>○ Sometimes<br>○ Less likely to<br>○ Never.	 70 (11.1%)<br>145 (23.1%)<br>187 (29.7%)<br>140 (22.3%)<br>87 (13.8%)
